# Predictive performance of dual modality of computed tomography angiography and intravascular ultrasound for no-reflow phenomenon after percutaneous coronary stenting in stable coronary artery disease

**DOI:** 10.1007/s00380-018-1160-2

**Published:** 2018-04-11

**Authors:** Masaaki Okutsu, Takeshi Horio, Hisataka Tanaka, Maki Akiyama, Niro Okimoto, Toshiyuki Tsubouchi, Kenji Kawajiri, Yasuhiro Ohashi, Satoru Sumitsuji, Yuji Ikari

**Affiliations:** 1 Department of Internal Medicine, Kawasaki Medical School General Medical Center, 2-6-1, Nakasange, Kita-ku, Okayama, 700-8505 Japan; 2 Department of Cardiology, Nozaki Tokushukai Hospital, Daito, Japan; 3 Department of General Internal Medicine, Nozaki Tokushukai Hospital, Daito, Japan; 4 Division of Cardiology for International Education and Research, Graduate School of Medicine, Osaka University, Suita, Japan; 5 Division of Cardiology, Tokai University School of Medicine, Isehara, Japan

**Keywords:** No-reflow, Percutaneous coronary intervention, Intravascular ultrasound, Computed tomography angiography, Plaque

## Abstract

Attenuated plaque on intravascular ultrasound (IVUS) and low attenuation plaque on computed tomography angiography (CTA) are associated with no-reflow phenomenon during percutaneous coronary intervention (PCI). However, evaluation by a single modality has been unable to satisfactorily predict this phenomenon. We investigated whether the combination of IVUS and CTA findings can ameliorate the predictive potential for no-reflow phenomenon after stent implantation during PCI in stable coronary artery disease (CAD). A total of 988 lesions of 707 stable CAD patients who underwent coronary CTA before PCI were enrolled. PCI was performed with preprocedural IVUS and stent implantation. As for plaque characters, very low attenuation plaque (CTA v-LAP) whose minimum density was < 0 Hounsfield units on CTA and attenuated plaque (IVUS AP) on IVUS were evaluated. No-reflow phenomenon was observed in 22 lesions (2.2%) of 19 patients (2.7%). Both CTA v-LAP and IVUS AP were much more frequently observed in patients with no-reflow phenomenon. Positive (PPV) and negative predictive values (NPV) and accuracy for prediction of no-reflow were almost equivalent between CTA v-LAP (13.2, 99.6, and 87.0%) and IVUS AP (15.7, 99.8, and 89.0%). The combination of CTA v-LAP and IVUS AP markedly ameliorated PPV (31.7%) without deterioration of NPV (99.7%) and increased the diagnostic accuracy (95.5%). These findings showed that the combination of CTA v-LAP and IVUS AP improved the predictive power for no-reflow phenomenon after coronary stenting in stable CAD patients, suggesting the usefulness of combined estimation by using CTA and IVUS for predicting no-reflow phenomenon during PCI in clinical practice.

**Electronic supplementary material:**

The online version of this article (10.1007/s00380-018-1160-2) contains supplementary material, which is available to authorized users.

## Introduction

No-reflow phenomenon that occurs through distal embolism derived from the plaque component after dilation procedures during percutaneous coronary intervention (PCI) causes transient myocardial ischemia. In many cases, it is reversible, but a reduction in blood flow is sometimes protracted, causing myocardial infarction and leading to an unfavorable prognosis [1–6]. It was reported that no-reflow phenomenon after dilation procedures occurred in the presence of marked lipid lesions, and that attenuated plaque observed on intravascular ultrasound (IVUS) was a predictive factor for this phenomenon [7–10]. However, in clinical practice, the positive predictive value of this factor is not high, and its predictive performance is controversial. Computed tomography angiography (CTA) with devices involving ≥ 64 channels provides favorable images [11–13]. Recently, it has been routinely used for coronary artery disease (CAD) screening. CTA facilitates the visualization of plaque in coronary vessel wall as well as stenosis of coronary lumen, and histological properties of plaque also can be diagnosed based on its CT density. Lipid-rich plaque visualized on CTA was reported to show a density of < 50 Hounsfield units (HU) [14]. In particular, plaque with low attenuation (a density of < 30 HU) was evaluated as thin cap fibrous atheroma in many cases [15] and was shown to contribute to the development of distal embolism during PCI or onset of acute coronary syndrome (ACS) [16–20]. However, no study has assessed the positive or negative predictive value of low-density plaque detected by CTA for the development of distal embolism during PCI. In addition, a single modality, IVUS or CTA, was used in previous studies regarding the prediction of distal embolism; no study has combined the two modalities. In this study, we evaluated the predictive performance for no-reflow phenomenon after stent implantation during PCI using a single modality, CTA or IVUS, in patients with clinically stable CAD, and examined whether the combination of CTA and IVUS findings improves the predictive power for this phenomenon.

## Materials and methods

### Subjects

Between May 2007 and March 2011, 988 lesions of 707 consecutively eligible patients with stable CAD who underwent coronary CTA and successful PCI with IVUS-guided stenting at the Department of Cardiology of the Nozaki Tokushukai Hospital were retrospectively enrolled in this study. All patients underwent PCI within 3 months (mean, 41 days) after coronary CTA. Lesions with chronic total occlusion, in-stent restenosis, Rotablator or excimer laser use, persistent major side branch occlusion jailed by stent, or nondiagnostic coronary CTA image and bypass graft lesion were excluded.

All study procedures were carried out in accordance with the institutional and national ethical guidelines for human studies. All of the patients gave informed consent to participation in this study. The study protocol was approved by the ethics committees of the Nozaki Tokushukai Hospital.

### Coronary CTA protocol

Coronary CTA examination was performed using Light Speed VCT (GE Healthcare, Milwaukee, WI). CT scan detector was 0.625 mm × 64 channels, rotation speed was 350 ms/rotation, helical pitch was 0.16–0.20, tube voltage was 120 kV, and current was 617 ± 96 (370–780) mA. Image was scanned with breath-holding and electrocardiogram (ECG) gating. For the contrast-enhanced scan, iohexol (Omunipaque 300, DAIICH SANKYO COMPANY, Tokyo, Japan) or iopamidol (Iopamiron 370, Bayer Yakuhin, Ltd, Osaka, Japan) was injected into the antecubital or forearm vein via a 20 or 22 G nylon needle. Injection volume was different according to body weight [0.7 or 0.8 mL × body weight (kg)] and the maximum dose was 50 mL. Contrast medium was injected at a rate of 3.0–6.5 mL/s according to body weight, followed by 20–40 mL saline. Before scanning, 15–20 mL contrast medium was injected as a test injection and the peak time of ascending aorta density was checked. Real scanning was started after this peak time from injection.

For pre-medication, 20 or 40 mg of metoprolol for patients having 60–70 bpm or more than 70 bpm, respectively, was administrated orally 2 h before CT scanning. When a patient had sinus rhythm and their heart rate was more than 65 bpm just before scanning, landiolol (12.5 mg) and/or propranolol (up to 12 mg) was injected intravenously. For the patients with atrial fibrillation, verapamil (up to 5 mg) as well as propranolol (up to 4 mg) was used for heart rate control at CT scanning.

### CT image analysis

**Fig. 1:**
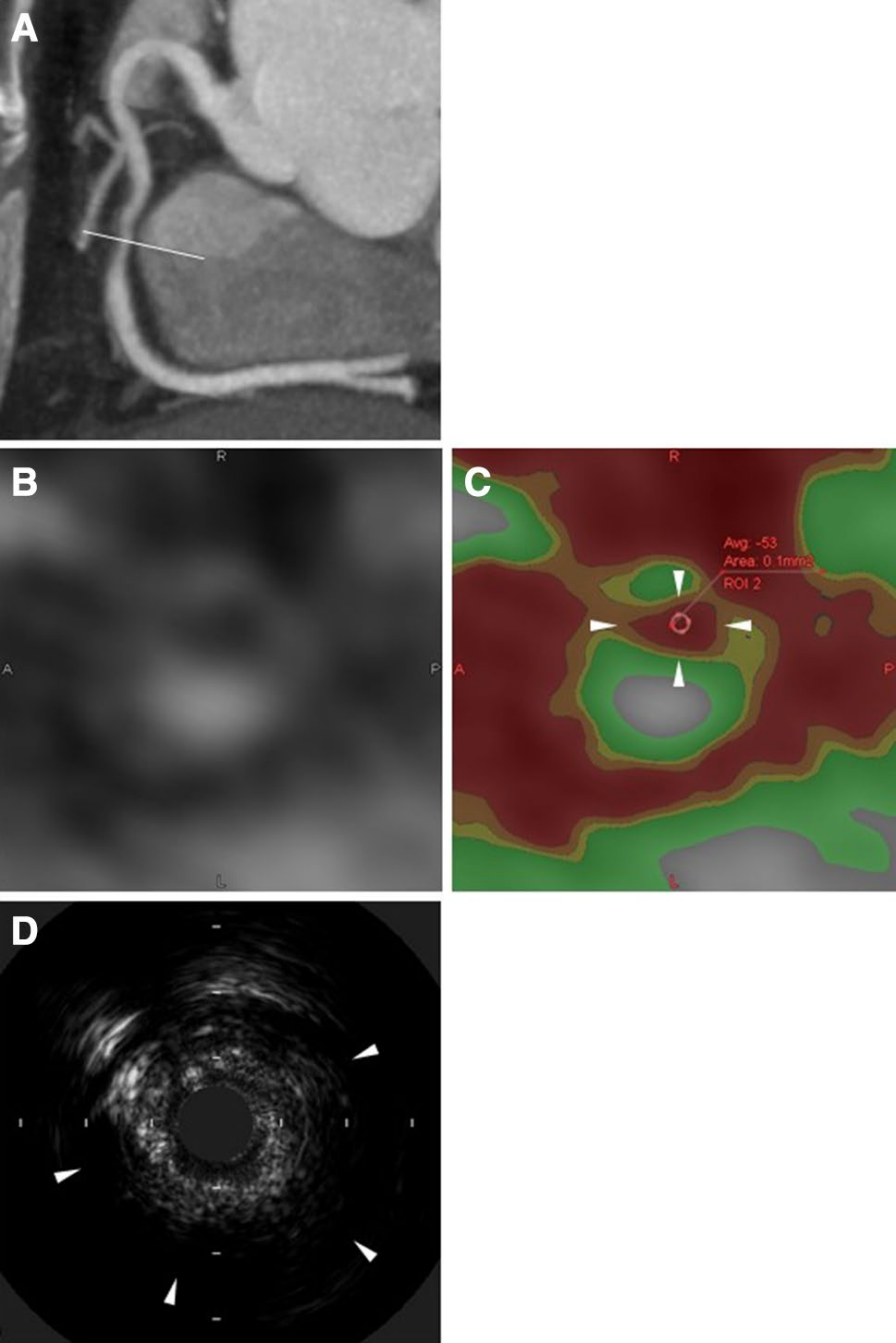
Representative images of CTA v-LAP (**a**–**c**) and IVUS AP (**d**). **a** Longitudinal coronary image and cross-sectional point by slab maximum intensity projection image. **b** Grayscale cross-sectional image. **c** Color map image and measurement of minimum CT density. Red area indicates < 0 HU density, i.e., v-LAP (arrowheads), orange area ≥ 0 and < 30 HU, yellow area ≥ 30 and < 50 HU, and green area ≥ 50 and < 250 HU. **d** Arrowheads indicate attenuated plaque on IVUS. *CTA* computed tomography angiography, *HU* Hounsfield units, *IVUS* intravascular ultrasound, *IVUS AP* attenuated plaque on IVUS, *v*-*LAP* very low attenuation plaque

The image was analyzed by the Zio station or zioTerm2009 (Ziosoft, Tokyo, Japan) with the Slab maximum intensity projection (MIP) method [21, 22]. Slab MIP method is one kind of image analyzing that is observed with changing slice angle, depth, and thickness of multiplanar reconstruction manually. Longitudinal image on Slab MIP method was observed with 5-mm thickness and cross-sectional image was with minimum thickness. Target lesion on CT was determined compared with coronary angiographic image. CT density was visualized by color map method classified with four levels (< 0 HU, ≥ 0 and < 30 HU, ≥ 30 and < 50 HU, ≥ 50 and < 250 HU). We defined plaque whose minimum density was < 30 HU as low attenuation plaque (LAP) and < 0 HU as very low attenuation plaque (v-LAP). A representative case with v-LAP is shown in Fig. 1a–c. If plaque of PCI target lesion had LAP or v-LAP, these areas were surveyed on cross-sectional image, and minimum CT density in the inside of LAP or v-LAP area was determined using average score within a small region of interest circle (about 0.2 mm^2^). Coronary calcification was classified into none, mild, moderate, and severe according to calcium arc on cross-sectional image. CT images were analyzed by one investigator who did not know PCI information.

### Angiographic and PCI protocol

Coronary angiography was performed using a bi-plane angiography system (DIGITEX-Safire SP HB, SHIMADZU CORPORATION, Kyoto, Japan) or single-plane angiography system (DIGITEX-Safire SP HC, SHIMADZU CORPORATION, Kyoto, Japan), and with 4 Fr. diagnostic catheter or guiding catheter (6 or 7 Fr.) and power injection system (ACIST CVi Injection system, ACIST Medical Systems, Minnesota, USA). As a contrast medium, iohexol (Omunipaque 350) or iopamidol (Iopamiron 370) was used. For coronary stent implantation, 100 mg/day aspirin and 200 mg/day ticlopidine or 75 mg/day clopidogrel were administrated orally from pre- or post-PCI if not contraindicated. Heparin (7000 U) was administered intravenously before PCI procedure and additional heparin was administered to keep activated clotting time > 250 s. PCI was performed with 6 or 7 Fr. guiding catheter. Stent diameter and length were decided from measurement by IVUS examination. Stent diameter was adjusted to reference lumen diameter and the plaque of the target lesion was fully covered by stent wherever possible. Indication of direct stenting without pre-balloon dilatation, indication of filter device, and whether drug eluting stent or bare metal stent should be used were decided by individual physicians. Post-balloon dilation was performed if stent dilation was not enough from IVUS measurement. Jailed major side branch was protected with guide wire before stent implantation. If side branch had severe stenosis or occlusion after stent implantation, additional balloon dilatation or kissing balloon inflation was performed.

### IVUS protocol

IVUS was performed before target lesion dilation and after stent implantation with 45 MHz Revolution (Volcano, California, USA), 40 MHz Atlantis Pro 2 (Boston Scientific, Massachusetts, USA), or 40 MHz Intrafocus II (Terumo, Tokyo, Japan). IVUS image was recorded on network server (Goodnet, Goodman, Aichi, Japan) using auto pullback (1 mm/s). Attenuated plaque was defined as intraplaque ultrasound attenuation within 3 mm from image core without calcification with distribution of more than 1 mm length and more than 180° of arc (Fig. 1d). IVUS images were analyzed by one investigator who did not know PCI information.

### Definition of no-reflow phenomenon

Coronary flow was assessed by one investigator who did not know CTA information. No-reflow phenomenon was defined as Thrombolysis In Myocardial Infarction (TIMI) flow grade ≤ 2, decreased coronary flow compared with pre-PCI angiogram, and ST segment elevation in ECG without visible coronary obstruction.

### Statistical analysis

Values are expressed as the mean ± SD. An unpaired *t* test was used for comparison of continuous variables between the two groups. Comparison of categorical variables between groups was performed by Chi square test. The sensitivity, specificity, positive predictive value (PPV), negative predictive value (NPV), and diagnostic accuracy of CTA and IVUS findings were calculated for prediction of no-reflow phenomenon. In all analyses, *p* < 0.05 was accepted as statistically significant.

## Results

### Patient characteristics

No-reflow phenomenon was observed in 19 (2.7%) of 707 patients. There was no statistically significant difference in age, gender, coronary risk factor, or previous PCI or coronary artery bypass graft between the two groups with and without no-reflow (Table 1). Creatinine kinase (CK)-MB elevation was defined as three times or more elevation than upper normal limit in the next day morning. If CK-MB level was not measured, CK was adopted instead of CK-MB. CK-MB (or CK) elevation was significantly more frequent in the no-reflow (+) group (15.8 vs. 1.6%, *p* < 0.001).

**Table 1:** Patient characteristics

	All patients	No-reflow (+)	No-reflow (−)	*p* value
	*n* = 707	*n* = 19	*n* = 688	
Age, years	68.6 ± 9.7	70.3 ± 9.6	68.5 ± 9.7	0.423
Male	486 (68.7)	15 (78.9)	471 (68.5)	0.331
Hypertension	572 (80.9)	15 (78.9)	557 (81.0)	0.826
Dyslipidemia	478 (67.6)	16 (84.2)	462 (67.2)	0.290
Diabetes mellitus	271 (38.3)	8 (42.1)	263 (38.2)	0.909
Smoking history	386 (54.6)	13 (68.4)	373 (54.2)	0.440
Hemodialysis	43 (6.1)	1 (5.3)	42 (6.1)	0.975
Previous PCI	331 (46.7)	10 (52.6)	320 (46.5)	0.598
Previous CABG	18 (2.5)	0 (0.0)	18 (2.6)	0.475
CK-MB (or CK) elevation	14 (2.0)	3 (15.8)	11 (1.6)	< 0.001

Values are mean ± SD or number (percentage)

*CABG* coronary artery bypass graft, *CK* creatinine kinase, *PCI* percutaneous coronary intervention

### Angiographic and procedural characteristics

The no-reflow phenomenon was observed in 22 (2.2%) of 988 lesions. As a target vessel for PCI, the right coronary artery was significantly more frequent and left circumflex artery was less frequent in the no-flow (+) group (Table 2). Although the final balloon size was not different between the two groups, maximum balloon pressure was significantly lower (14.4 ± 4.0 atm vs. 16.5 ± 4.3 atm, *p* = 0.007) and the use of filter device for distal protection was more frequent (22.7 vs. 0%, *p* < 0.001) in the no-reflow (+) group.

**Table 2:** Angiographic and procedural characteristics

	All lesions	No-reflow (+)	No-reflow (−)	*p* value
	*n* = 988	*n* = 22	*n* = 966	
Target vessel				
RCA	279 (28.2)	12 (54.5)	267 (27.6)	0.006
LMT	34 (3.4)	1 (4.5)	33 (3.4)	0.774
LAD	423 (42.8)	8 (36.4)	415 (43.0)	0.536
LCX	252 (25.5)	1 (4.5)	251 (26.0)	0.023
Final balloon size, mm	2.94 ± 0.48	3.10 ± 0.43	2.94 ± 0.47	0.117
Maximum pressure, atm	16.4 ± 4.3	14.4 ± 4.0	16.5 ± 4.3	0.007
Filter device use	5 (0.5)	5 (22.7)	0 (0.0)	< 0.001

Values are mean ± SD or number (percentage)

*LAD* left anterior descending artery, *LCX* left circumflex artery, *LMT* left main trunk, *RCA* right coronary artery

### CTA and IVUS findings

The prevalence rates of low-density plaque on CTA, which was defined as three different cutoff values of minimum CT density, i.e., less than 0 HU, 30 HU, and 50 HU, were 144 (14.6%), 210 (21.3%), and 250 (25.3%), respectively (Table 3). On the other hand, the prevalence of attenuated plaque evaluated by IVUS (IVUS AP) was 127 (12.9%). As expected, the presence of IVUS AP was closely associated with the existence of low-density plaque on CTA (Supplementary Table S1).

**Table 3:** CTA and IVUS findings

	All lesions	No-reflow (+)	No-reflow (−)	*p* value
	*n* = 988	*n* = 22	*n* = 966	
CTA				
Minimum CT density				
< 0 HU (v-LAP)	144 (14.6)	19 (86.4)	125 (12.9)	< 0.001
< 30 HU (LAP)	210 (21.3)	21 (95.5)	189 (19.6)	< 0.001
< 50 HU	250 (25.3)	21 (95.5)	229 (23.7)	< 0.001
Calcified lesion	608 (61.5)	11 (50.0)	597 (61.8)	0.261
Calcium level				
Mild	308 (31.2)	4 (18.2)	304 (31.5)	0.183
Moderate	195 (19.7)	6 (27.3)	189 (19.6)	0.369
Severe	105 (10.6)	1 (4.5)	104 (10.8)	0.349
IVUS				
IVUS AP	127 (12.9)	20 (90.9)	107 (11.1)	< 0.001

Values are number (percentage)

*CTA* computed tomography angiography, *HU* Hounsfield units, *IVUS* intravascular ultrasound, *IVUS AP* attenuated plaque on IVUS, *LAP* low attenuation plaque, *v*-*LAP* very low attenuation plaque

Comparison of the CTA and IVUS findings between the two groups with and without no-reflow phenomenon, low-density plaque detected by CTA was more frequently observed in the no-reflow (+) group, regardless of the three different cutoff values of minimum CT density (< 0 HU, 86.4 vs. 12.9%, *p* < 0.001; < 30 HU, 95.5 vs. 19.6%, *p* < 0.001; < 50 HU, 95.5 vs. 23.7%, *p* < 0.001) (Table 3). Similarly, IVUS AP was significantly frequent in the no-reflow (+) group (90.9 vs. 11.1%, *p* < 0.001). There was no difference in the presence of calcified lesion or calcium level between the two groups.

### Prediction of no-reflow phenomenon

The prevalence rates of no-reflow phenomenon in three subgroups with low-density plaque on CTA, i.e., with < 0 HU, < 30 HU, and < 50 HU of minimum CT density, were 13.2, 10.0, and 8.3%, respectively (Supplementary Table S2). When several predictive indices for no-reflow phenomenon in the three subgroups were examined, PPV and accuracy were highest in the subjects with < 0 HU of minimum CT density, that is, with v-LAP (Supplementary Table S3).

The associations of the existence of CTA v-LAP and IVUS AP with no-reflow phenomenon are shown in Table 4. The CTA v-LAP (+) group had significantly more no-reflow phenomenon than the CTA v-LAP (−) group (13.2 vs. 0.4%, *p* < 0.001). Similarly, the IVUS AP (+) group had significantly more no-reflow phenomenon than the IVUS AP (−) group (15.7 vs. 0.2%, *p* < 0.001). The group with both CTA v-LAP and IVUS AP also had significantly more no-reflow phenomenon than the other one (31.7 vs. 0.3%, *p* < 0.001), and the prevalence of no-reflow phenomenon was clearly elevated by the combination of CTA v-LAP and IVUS AP.

**Table 4:** Prevalence of no-reflow phenomenon according to CTA v-LAP and IVUS AP

	CTA			IVUS			CTA and IVUS		
	v-LAP (+)	v-LAP (−)	*p* value	AP (+)	AP (−)	*p* value	v-LAP (+) and AP (+)	v-LAP (−) or AP (−)	*p* value
	*n* = 144	*n* = 844		*n* = 127	*n* = 861		*n* = 60	*n* = 928	
No-reflow (−)	125 (86.8)	841 (99.6)	< 0.001	107 (84.3)	859 (99.8)	< 0.001	41 (68.3)	925 (99.7)	< 0.001
No-reflow (+)	19 (13.2)	3 (0.4)		20 (15.7)	2 (0.2)		19 (31.7)	3 (0.3)	

Values are number (percentage)

*AP* attenuated plaque, *CTA* computed tomography angiography, *IVUS* intravascular ultrasound, *v*-*LAP* very low attenuation plaque

The sensitivity, specificity, PPV, NPV, and accuracy of CTA v-LAP for prediction of the no-reflow phenomenon were 86.4, 87.1, 13.2, 99.6, and 87.0%, respectively (Table 5). Likewise, those of IVUS AP were 90.9, 88.9, 15.7, 99.8, and 89.0%, respectively. When evaluating the predictive value of the combination of CTA v-LAP and IVUS AP, its sensitivity, specificity, PPV, NPV, and accuracy for the no-reflow phenomenon were 86.4, 95.8, 31.7, 99.7 and 95.5%, respectively, suggesting that the combination of these two factors improved PPV and accuracy without decreasing NPV.

**Table 5:** Prediction of no-reflow phenomenon by CTA v-LAP and IVUS AP

	Sensitivity (%)	Specificity (%)	PPV (%)	NPV (%)	Accuracy (%)
CTA v-LAP	86.4	87.1	13.2	99.6	87.0
IVUS AP	90.9	88.9	15.7	99.8	89.0
CTA v-LAP + IVUS AP	86.4	95.8	31.7	99.7	95.5

*CTA v*-*LAP* very low attenuation plaque on computed tomography angiography, *IVUS AP* attenuated plaque on intravascular ultrasound, *NPV* negative predictive value, *PPV* positive predictive value

## Discussion

We evaluated each predictive performance of IVUS or CTA for the development of no-reflow phenomenon after stenting in patients with stable CAD, and examined for the first time whether the combination of the two modalities improved the predictive performance for this phenomenon. IVUS AP was observed in 12.9% of the lesions and CTA v-LAP in 14.6%. No-reflow phenomenon occurred in 2.2%. The negative predictive values of IVUS AP and CTA v-LAP were extremely favorable, but the positive predictive values of the respective modalities were not sufficient. On the other hand, no-reflow phenomenon appeared in 31.7% of lesions with both IVUS AP and CTA v-LAP, facilitating favorable positive prediction. The accuracy further improved to 95.5%. In addition, the NPV of either finding was 99.7%; there was no deterioration in comparison with the NPVs of the respective findings as a single modality.

Previous studies involving patients with ACS lesions or a population consisting of patients with ACS and non-ACS lesions predicted no-reflow phenomenon using IVUS or CTA. However, thrombi may be present in ACS lesions, and thrombus-related distal embolism may be included; the accuracy of prediction based on plaque assessment on IVUS or CTA may be reduced. Therefore, in this study, the subjects were limited to patients with stable CAD to evaluate the prediction of plaque-related distal embolism, excluding thrombus formation as an etiological factor.

Some previous studies showed that attenuated plaque on IVUS was associated with the no-reflow phenomenon during PCI or myocardial damage after PCI [7, 8, 23, 24]. However, few studies have reported its predictive performance. Lee et al. [25] reported that, in patients with ACS, the coronary flow after PCI was TIMI flow grade ≤ 2 in 26.7% of culprit lesions with attenuated plaque, while TIMI flow grade 3 was observed after PCI in 95.4% of those without such plaque. This study involved patients with stable CAD, and the incidence of no-reflow phenomenon was low; therefore, the PPV was 15.7% based on IVUS findings, whereas the accuracy was 89.0% due to extremely high NPV (99.8%). When predicting accidents in clinical practice, not only a statistically significant correlation, but also high NPV are required; therefore, IVUS is useful for predicting the development of the no-reflow phenomenon. However, the PPV is low; concerning this issue, the clinical usefulness of IVUS is not sufficient.

On the other hand, previous studies regarding CTA reported that the minimum CT density of plaque was significantly lower in patients with the no-reflow phenomenon [26, 27]. Kinohira et al. [28] showed that the occurrence of transient slow flow was not rare during PCI in lesions with soft plaque whose minimum CT density was < 50 HU. Uetani et al. [29] also showed that the volume of < 50 HU within target lesions had an association with post-procedural elevation of cardiac damage-related biomarker levels. Harigaya et al. [16] reported that the longitudinal length of LAP (CT density: < 30 HU) was significantly correlated with the no-reflow phenomenon. However, these studies did not investigate its predictive performance. In this study, LAP, which was previously reported as a predictive factor for the no-reflow phenomenon, was observed in 21.3% of all the lesions, and the NPV was 99.9%. However, the PPV was low (10.0%), with an accuracy of 80.8%. On the other hand, the PPV, NPV, and accuracy of v-LAP (minimum CT density: < 0 HU), which we defined in this study, were 13.2, 99.6, and 87.0%, respectively, exceeding those of LAP. However, its positive prediction was not sufficient, as described for prediction based on attenuated plaque on IVUS.

The reasons why the PPV of attenuated plaque on IVUS for the no-reflow phenomenon was low include the presence of plaque with pathological intimal thickness, stable early necrotic core, and microcalcification in IVUS AP [30–32]. The reasons why the PPV of v-LAP on CTA for the no-reflow phenomenon was low include the presence of artifacts, including a beam-hardening artifact [33]. For this reason, a CT density differing from the CT density that tissue should essentially have is indicated, reducing the preciseness of prediction.

The PPV of plaque with two findings: attenuated plaque on IVUS and v-LAP on CTA was 31.7%, showing a marked improvement. The NPV in the absence of either finding was 99.7%, being extremely favorable. The PPV and NPV may be at a clinically useful level. The accuracy of IVUS for visualizing findings other than calcification is high, and this procedure may cover the artifact-related inaccuracy of CTA. CTA facilitates the more accurate assessment of plaque lipid properties, which are difficult to distinguish on IVUS. Thus, the two procedures may have improved the accuracy of predicting the no-reflow phenomenon by covering each other’s disadvantages. The use of two diagnostic modalities, CTA as a routine examination to detect coronary artery lesions, and IVUS as a routine supporting tool for PCI, facilitates the accurate prediction of the no-reflow phenomenon, which is clinically significant.

This study has the following limitations. (1) This was a single-center, retrospective observational study involving stable CAD patients who had undergone elective PCI; the incidence of events (no-reflow phenomenon) was relatively low. (2) As detailed data of medication for the present subjects were lacking, we could not rule out the possibility that medical treatment such as antiplatelet therapy and statin use before PCI had biased our results. (3) PCI operators had checked CTA images before PCI and also performed IVUS just before PCI; these kinds of information might influence the decision of maximum dilation pressure and filter device use, particularly for expected high-risk lesions. (4) CTA showed physical status-/endovascular contrast medium concentration-/calcification-related differences in the CT density of plaque, but they were not corrected. (5) Plaque property assessment using CTA was based on the CT density on a single cross section; the volume of low CT density plaque involving a longitudinal direction was not evaluated. Furthermore, neither arc nor length of attenuated plaque on IVUS was quantitatively assessed. (6) No other predictive factor for no-reflow phenomenon on CTA, as previously reported, such as positive remodeling or the presence of napkin-ring signs [24, 25], was evaluated.

## Conclusion

The predictive performance of the combination of CTA and IVUS for the no-reflow phenomenon after coronary stenting in patients with stable CAD was more accurate than that of a single modality. In this method, imaging findings are evaluated based on the presence or absence of CTA v-LAP and IVUS AP alone; the no-reflow phenomenon can be predicted simply and accurately. The predictive performance does not solely indicate the correlation between CTA/IVUS findings and no-reflow phenomenon, but it may be useful for predicting the development of this phenomenon during PCI in clinical practice.

## Electronic supplementary material

Below is the link to the electronic supplementary material.

Supplementary material 1 (DOCX 20 kb)
