# Supplementary material for: Predictive performance of dual modality of computed tomography angiography and intravascular ultrasound for no-reflow phenomenon after percutaneous coronary stenting in stable coronary artery disease
Source: Heart Vessels. 2018 Apr 11;33(10):1121–8. doi: 10.1007/s00380-018-1160-2 (PMC6133068; doi:10.1007/s00380-018-1160-2)
Supplement: Supplementary file 1 — Supplementary material 1 (DOCX 20 kb) [file 380_2018_1160_MOESM1_ESM.docx]

**Supplementary Table S1** Prevalence of attenuated plaque on IVUS according to minimum CT density in the plaque

　　 Minimum CT density

<0 HU ≥0 HU <30 HU ≥30 HU <50 HU ≥50 HU

n=144 n=844 p Value n=210 n=778 p Value n=250 n=738 p Value

IVUS AP (-) 84 (58.3) 777 (92.1) 129 (61.4) 732 (94.1) 157 (62.8) 704 (95.4)

IVUS AP (+) 60 (41.7) 67 (7.9) <0.001 81 (38.6) 46 (5.9) <0.001 93 (37.2) 34 (4.6) <0.001

Values are number (percentage)

*CT* computed tomography, *HU* Hounsfield units, *IVUS* intravascular ultrasound, *IVUS AP* attenuated plaque on IVUS

**Supplementary Table S2** Prevalence of no-reflow phenomenon according to minimum CT density in the plaque

　　 Minimum CT density

<0 HU ≥0 HU <30 HU ≥30 HU <50 HU ≥50 HU

n=144 n=844 p Value n=210 n=778 p Value n=250 n=738 p Value

No-reflow (-) 125 (86.8) 841 (99.6) 189 (90.0) 777 (99.1) 229 (91.7) 737 (99.9)

No-reflow (+) 19 (13.2) 3 (0.4) <0.001 21 (10.0) 1 (0.1) <0.001 21 (8.3) 1 (0.1) <0.001

Values are number (percentage)

*CT* computed tomography, *HU* Hounsfield units

**Supplementary Table S3** Prediction of no-reflow phenomenon by CT density in the plaque

Sensitivity (%) Specificity (%) PPV (%) NPV (%) Accuracy (%)

<0 HU (v-LAP) 86.4 87.1 13.2 99.6 87.0

<30 HU (LAP) 95.5 80.4 10.0 99.9 80.8

<50 HU 95.5 76.3 8.4 99.9 73.8

*CT* computed tomography, *HU* Hounsfield units, *LAP* low attenuation plaque, *NPV* negative predictive value, *PPV* positive predictive value, *v-LAP* very low attenuation plaque
